# Reconstructing ecosystem functions of the active microbial community of the Baltic Sea oxygen depleted sediments

**DOI:** 10.7717/peerj.1593

**Published:** 2016-01-19

**Authors:** Petter Thureborn, Andrea Franzetti, Daniel Lundin, Sara Sjöling

**Affiliations:** 1 School of Natural Sciences, Technology and Environmental Studies, Södertörn University, Huddinge, Sweden; 2 Department of Earth and Environmental Sciences, University of Milano-Bicocca, Milano, Italy; 3 BILS, Bioinformatics Infrastructure for Life Sciences, Science for Life Laboratories, Solna, Sweden; 4 Centre for Ecology and Evolution in Microbial model Systems—EEMiS, Linnaeus University, Kalmar, Sweden

**Keywords:** Baltic Sea, Microbial functions, Eutrophication, Anoxic sediment, Oxygen depletion, Cyanobacteria, Integron integrase, Metatranscriptome, Methanogenesis, Methane oxidation

## Abstract

Baltic Sea deep water and sediments hold one of the largest anthropogenically induced hypoxic areas in the world. High nutrient input and low water exchange result in eutrophication and oxygen depletion below the halocline. As a consequence at Landsort Deep, the deepest point of the Baltic Sea, anoxia in the sediments has been a persistent condition over the past decades. Given that microbial communities are drivers of essential ecosystem functions we investigated the microbial community metabolisms and functions of oxygen depleted Landsort Deep sediments by metatranscriptomics. Results show substantial expression of genes involved in protein metabolism demonstrating that the Landsort Deep sediment microbial community is active. Identified expressed gene suites of metabolic pathways with importance for carbon transformation including fermentation, dissimilatory sulphate reduction and methanogenesis were identified. The presence of transcripts for these metabolic processes suggests a potential for heterotrophic-autotrophic community synergism and indicates active mineralisation of the organic matter deposited at the sediment as a consequence of the eutrophication process. Furthermore, cyanobacteria, probably deposited from the water column, are transcriptionally active in the anoxic sediment at this depth. Results also reveal high abundance of transcripts encoding integron integrases. These results provide insight into the activity of the microbial community of the anoxic sediment at the deepest point of the Baltic Sea and its possible role in ecosystem functioning.

## Introduction

Globally, oxygen depleted areas of seas and oceans have increased over the past 50 years (Diaz & Rosenberg, 2008; Stramma et al., 2008). In the Baltic Sea the hypoxic areas have expanded from 5,000 km^2^ to 60,000 km^2^ over the past century mainly due to eutrophication (Carstensen et al., 2014a). The expansion of hypoxia has escalated in recent years (Zillén & Conley, 2010; Hansson & Andersson, 2014) resulting in some of the largest oxygen depleted and sulphidic areas in the world (Diaz & Rosenberg, 2008). This is specifically evident in the Baltic Proper, the central part of the Baltic Sea, a brackish water basin with limited water ventilation and high nutrient load from agricultural production, riverine input, and seasonal phytoplankton blooms (Conley et al., 2009). Microbial respiration of organic matter, e.g., from the phytoplankton blooms, effectively depletes dissolved oxygen below the halocline (Cederwall & Elmgren, 1990), resulting in hypoxic waters and anoxic sediments (Cederwall & Elmgren, 1990; Diaz & Rosenberg, 2008; HELCOM, 2009). Consequences of oxygen depletion are changes in the biogeochemical cycles of nutrients and ecosystem energy flows (Conley et al., 2009; Carstensen et al., 2014b), in which microbial populations have an essential function (Falkowski, Fenchel & Delong, 2008). Ultimately, severe oxygen deficiency will increase mortality of macro-benthic organisms, which leads to deterioration of benthic communities and fish habitat of the Baltic Sea ecosystem (Cederwall & Elmgren, 1990; Conley et al., 2009; Carstensen et al., 2014b). This results in what are called ‘dead zones’ (Diaz & Rosenberg, 2008) with both ecological and economic consequences (Elmgren, 2012; Ahtiainen et al., 2014).

The euxinic conditions are especially pronounced at the highly stratified Landsort Deep that stretches into the deepest point (466 m) of the Baltic Sea. At depth, anoxia and sulphide have been persistent features for the past decade (SMHI, 2014) and the lamination of Landsort Deep bottom sediment suggests essentially anoxic conditions at the sediment surface during the past ca. 4500 ^14^C years (Lepland & Stevens, 1998). The sediment at this site is rich in organic material since flocks of cells, from phytoplankton blooms and other organic particles, sink through the water column (Leipe et al., 2011) and accumulate at the sediment surface, resulting in so called accumulation bottoms (Fredén, 1994). Particulate organic carbon values of the Baltic Proper deep are comparable only to regions of extreme productivity and oxygen depletion, such as the upwelling areas off Peru, SW Africa and the Arabian Sea (Leipe et al., 2011 and references therein). Moreover, the Baltic Sea is affected by anthropogenic pollution, heavy metals and organic pollutants have accumulated in the anoxic sediment of the Landsort Deep (HELCOM, 2010).

Transformation of organic compounds and nutrients is largely driven by microbial communities. Through a metagenomic survey of microbial communities along the oxygen gradient at Landsort Deep, we previously showed that there was a functionally and taxonomically diverse bacterial and archaeal community in the sediment (Thureborn et al., 2013). While a metagenomic survey reveals the functional capacities it does not disclose activity and if the genomic content is transcribed. Since DNA may stay preserved at anoxic conditions (Corinaldesi et al., 2011) it cannot be excluded that the metagenome represents sedimented possibly dead cells rather than a local functionally active community. Recent studies have analysed the taxonomic composition of Baltic Proper microbial communities in oxygen depleted sediments rich in organic compounds (Sinkko et al., 2013) and surveyed specific microbial functions e.g., methane oxidation or acetate uptake, in the hypoxic pelagic zone (Berg et al., 2013; Jakobs et al., 2013). However, what functions of the microbial community that are expressed as gene transcripts in the sediment of the ‘Baltic Sea dead zone’ and how the sediment community at Landsort Deep *in situ* capitalise on the organic matter in the anoxic environment have not previously been investigated. Recently a metatranscriptomic experimental study of sulphidic marine sediments from methane seeps near Barbados and from the Santa Barbara Basin showed differences in polyphosphate metabolism at oxic and anoxic conditions (Jones, Flood & Bailey, 2015). However, *in situ* microbial community transcriptional activity of deep sea marine sediments in general is poorly described since there are only two published studies to date, i.e., metatranscriptomes from Peru Margin sub seafloor (Orsi et al., 2013) and Arctic Jan Mayen vent field (Urich et al., 2014). No metatranscriptomics studies have focused on anoxic brackish sediments. With the prospect of a progression of oxygen depletion both in the Baltic Proper (Meier et al., 2011; Kabel et al., 2012) and globally (Altieri & Gedan, 2015), a deeper understanding of microbial metabolic processes in anoxic sediments is increasingly important as these processes are key in essential ecosystem functions such as carbon, sulphur and nitrogen transformation.

In this study, we investigated what functional capacities of the microbial community in the Landsort Deep anoxic sediment were realised, i.e., actually expressed, using a metatranscriptomics approach. We analysed the expression of metabolic and regulatory pathways to elucidate the ecosystem functions that the sediment microbial community potentially contributes to. Given the eutrophication process with extension of anoxic zones in the Baltic Sea and the global importance of carbon cycling, we specifically considered sediment community transformation of organic carbon. The results reveal a sediment microbial community with active expression of genes for essential nutrient transformation processes and provide metatranscriptomic information on the deepest part of the Baltic Sea. The information is important for understanding microbial ecosystem functions of the expanding anoxic sediment areas of the Baltic Sea but also pertinent in the wider perspective of eutrophied marine environments and anoxic sediments.

## Materials and Methods

### Sampling

Triplicate sediment cores for nucleic acid extraction and elemental analysis were retrieved at 466 m depth, using a Gemini sampler, on the 21st of April 2010 at Landsort Deep (lat 583591N, long 01814.26E) in the Baltic Sea, Sweden. Measurements and analyses of environmental parameters and nutrients were performed as previously described (Thureborn et al., 2013).

### RNA and DNA extraction

Nucleic acids were extracted from the 0–10 cm top-layer of triplicate sediment cores. Total RNA extraction, using the FastRNA Pro Soil-Direct Kit (MP Biomedicals, Solon, OH, USA), was immediately initiated on board the ship. 500 mg sediment aliquots were immersed in RNApro Soil Lysis Solution and processed in the FastPrep instrument followed by transportation on dry ice to the lab and storage at −80°C. The extraction procedure continued in the lab following the manufacturer’s instructions. Total RNA extracts were treated with Turbo DNA-free kit (Life Technologies, New York, NY, USA) and DNAseI (Thermo Fisher Scientific, Waltham, MA, USA) according to the manufacturers’ protocols. The RNA was quantified and RNA integrity was analysed using a Qubit fluorometer (Life Technologies) and Bioanalyzer (Agilent Technologies, Santa Clara, CA, USA), respectively. Total DNA was extracted, as described previously (Thureborn et al., 2013), and subsequently pooled before sequencing.

### cDNA synthesis

Prior to synthesis of cDNA, total RNA extracts from the triplicate sediment cores were pooled and subsequently split into two aliquots. One of the aliquots was enriched for mRNA using the MICROBE *Express* Bacterial mRNA Enrichment Kit (Life Technologies). The two fractions, i.e., the mRNA enriched and the total RNA, were amplified separately using the MessageAmp II Bacteria kit (Life Technologies) following the manufacturer’s instructions, except substituting the kit oligo (dT) with T7-BpmI-(dT)^16^ VN (Frias-Lopez et al., 2008). The antisense RNA product was synthesised into cDNA using SuperScript II Reverse Transcriptase (Life Technologies) with random hexamer priming for first-strand synthesis and Superscript Double Stranded cDNA synthesis kit (Life Technologies) for second-strand synthesis following the manufacturer’s instructions. The synthesised cDNA was purified using the Qiaquick PCR Purification Kit (Qiagen, Hilden, Germany) and subsequently treated with 2–3 units *Bpm*I (New England Biolabs, Ipswich, MA, USA) per µg cDNA for 2–3 h at 37 °C to remove poly-A tails. The *Bpm*I treated cDNA was purified using Qiaquick PCR Purification Kit before being sent to sequencing.

### Sequencing

The two cDNA fractions (i.e., total cDNA and mRNA-enriched cDNA) were sequenced as 100 bp paired-end reads on a third of a lane, respectively, on an Illumina HiSeq 2000 (Illumina Inc, San Diego, CA, USA) at the Science For Life Laboratory (SciLifeLab), Stockholm, Sweden. The DNA was sequenced as 100 bp paired-end reads on one lane on an Illumina HiSeq 2000 (Illumina Inc) at GATC Biotech AG, Konstanz, Germany. In total, the Illumina sequencing generated approximately 187 million cDNA and 106 million DNA paired-end sequence reads (2 × 100 bp), respectively (Table S1).

Sequence data were deposited at the European Nucleotide Archive (ENA) under the project PRJEB6616 with sample accession numbers ERS485200 (total cDNA), ERS485201 (mRNA-enriched cDNA) and ERS485202 (DNA).

### Bioinformatic analyses

Quality check of the Landsort Deep sediment cDNA and DNA paired-end sequence reads was performed using FastQC version 0.11.2 (http://www.bioinformatics.babraham.ac.uk/projects/fastqc/). Prior to further bioinformatics analyses, the cDNA and DNA paired-end sequence reads and those retrieved from published sediment metatranscriptomes (Orsi et al., 2013; Urich et al., 2014) were quality-trimmed (minimum length: 80 bp; average quality score: 30) using Sickle (https://github.com/najoshi/sickle). Ribosomal RNA (rRNA) sequences were removed from all cDNA datasets using ERNE-FILTER (Prezza et al., 2012) with sequences from the ARB SILVA LSU and SSU databases (release 111) (Quast et al., 2013). The two Landsort Deep cDNA datasets (i.e., total cDNA and mRNA-enriched cDNA) showed similar results in terms of quality trimming and removal of rRNA sequences. Prior to assembly the sequence reads from these two cDNA datasets were merged, generating one cDNA dataset. The Landsort Deep cDNA and DNA paired-end sequence reads were assembled separately using the Velvet (with Oases in case of cDNA) and Meta-Ray assemblers (Zerbino & Birney, 2008; Boisvert et al., 2012; Schulz et al., 2012). For cDNA assembly, the following kmer lengths were used: 57, 61, 65, 69 with Velvet-Oases and 33, 41, 49, 57, 61, 65 with Meta-Ray. For DNA assembly the following kmer lengths were used: 51, 55 with Velvet and 31 with Meta-Ray. Genes were predicted from the obtained contigs using FragGeneScan with suggested options for contigs (Rho, Tang & Ye, 2010) generating 429,162 cDNA genes and 3,176,262 DNA genes, respectively (Table S1). The predicted gene sequences obtained with the different assemblers and kmer lengths were clustered at 99% similarity using UCLUST (Edgar, 2010). Representative sequences from each cluster were retrieved for further processing. The obtained sequences were aligned to the M5NR database (release 20120401) (Wilke et al., 2012) using BLASTX (Altschul et al., 1997). The BLASTX outputs, with a bit score >50, were used to annotate ORFs in KEGG (Kanehisa et al., 2012) and SEED (Overbeek et al., 2005) categories, and the Last Common Ancestor (LCA) algorithm (Huson et al., 2007) was applied to taxonomically bin each sequence using in-house scripts (m5nr2annot and organisms2lca in https://github.com/erikrikarddaniel/environmentmicrobedb-tools). The LCA algorithm, as run by us, considers the taxonomy of all BLASTX hits with a bit score >50 and >0.9 times the bit score of the best hit. The taxonomy assigned to a sequence will be the most specific that is common to all BLASTX hits. The estimation of the relative abundance of each SEED/KEGG function or category was obtained by mapping the quality-trimmed sequence reads against the annotated ORFs using ERNE-MAP (Prezza et al., 2012). Ultimately, around 5 million cDNA and 12 million DNA sequence reads could be annotated to a SEED function (Table S1).

Phylogenetic analysis was performed to reveal if methyl coenzyme M reductase A (*mcrA)* transcripts were affiliated to methanogenic or anaerobic methane oxidising (ANME) archaea. These transcripts were aligned to selected *mcrA* genes from methanogenic and ANME (Hallam et al., 2003; Lösekann et al., 2007) archaea using ClustalW (Thompson, Higgins & Gibson, 1994) as implemented in Geneious version (5.0.4) (Kearse et al., 2012) and subsequently analysed using the Neighbor-Net (Bryant & Moulton, 2004) algorithm with uncorrected p-distances implemented in SplitsTree4 (Huson & Bryant, 2006). The contig containing the single ORF that encoded beta-lactamase was aligned against the NCBI non-redundant (NR) database using BLASTX. To reveal any possible contamination the beta-lactamase ORF was further aligned to commercial cloning- and expression vectors used in our lab using ClustalW in Geneious. Transcripts encoding the D1 protein of the PSII P680 reaction centre (*psbA*) were taxonomically assigned using BLASTX against the NCBI NR database and MEGAN (Huson et al., 2011) with best hit as criteria. To reveal if there was a diatom signature in the rRNA, the sequence reads from the non-enriched cDNA fraction (i.e., total cDNA) were annotated using the MG-RAST pipeline and the integrated SSU and LSU rRNA databases (Meyer et al., 2008).

The Landsort Deep sediment data was compared to metatranscriptomes from the Arctic Jan Mayen vent field (Urich et al., 2014) and Peru Margin sub-seafloor (Orsi et al., 2013) sediments. The quality-trimmed reverse (3′) reads from the mRNA-enriched Landsort Deep, the forward (5′) reads from total RNA Landsort Deep and Peru Margin (5, 30, 70 and 159 m below the sea floor) paired-end Illumina datasets and the 454 reads from Arctic Jan Mayen vent field were aligned to the NCBI NR database using DIAMOND (Buchfink, Xie & Huson, 2015). DIAMOND output files were imported into MEGAN5 (Huson et al., 2011) where metatranscriptomic sequence reads were annotated to SEED functions (Overbeek et al., 2005) using the default settings. Comparison of the different datasets was performed using sub-sampled counts.

### Data analysis

For identification and visualisation of regulatory and metabolic pathways in KEGG, iPATH_2_ (Yamada et al., 2011) was used. All KEGG identifiers (i.e., KO numbers) with ≥1 hit in the metatranscriptomic dataset were applied. Prior to analysis of SEED annotated data, the sequences for functional role *Retron-type reverse transcriptase* were removed from the cDNA and DNA datasets because of suspicious misannotation of rRNA (Tripp et al., 2011). In the hierarchical SEED functional category system several functions (i.e., proteins) are affiliated to multiple SEED categories. Consequently when summarising the number of reads at the higher hierarchical levels (i.e., 1st and 2nd level of SEED) there is a risk that the same reads are counted more than once. To avoid this we removed duplicate proteins affiliated to the same parent SEED category when summarising number of reads at the 1st and 2nd level of the SEED hierarchy. The portion of functions (i.e., genes) expressed within functional categories (i.e., SEED categories) was calculated as the ratio of functions detected in the metatranscriptome/functions detected in the metagenome. Relative abundances of taxa and functions were calculated as percentage of the total number of reads annotated in the metatranscriptome(s) and metagenome, respectively.

## Results

### Environmental parameters

At the time of sampling, the water overlying the sediment was depleted of dissolved oxygen and hydrogen sulphide (H_2_S) (16.3 µmol/L) was present (Table S2). Measurements of sediment nutrients revealed high concentrations of ammonium (363.8 µmol/L), phosphate (46.9 µmol/L) and silica (268.2 µmol/L), and low concentration of nitrate (4.1 µmol/L) (Table S2). The sediment total carbon content (TOC) was approximately 10% and the concentration of dissolved organic carbon (DOC) was 79.0 mg/L (Table S2).

### Active gene expression of metabolic and regulatory pathways of the sediment community

Annotation of the gene transcripts present in the Landsort Deep sediment metatranscriptome revealed that as much as 30% of the total functions annotated in the corresponding metagenome were expressed. The major part, 99%, of the functions identified in the metatranscriptome could correspondingly be confirmed in the metagenome.

**Figure 1:**
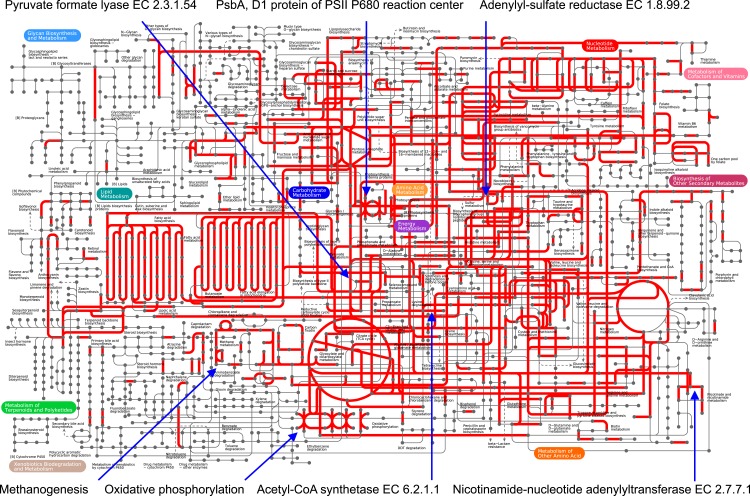
Expression of metabolic genes by the Landsort Deep sediment community. Overview of all metabolic KEGG pathways expressed as transcripts (RNA) by the total microbiome in the Landsort Deep sediment. Mapping of transcript data to the KEGG map was performed using iPATH v2 (Yamada et al., 2011). Red indicates transcript (element) present in the Landsort Deep sediment metatranscriptome.

**Figure 2:**
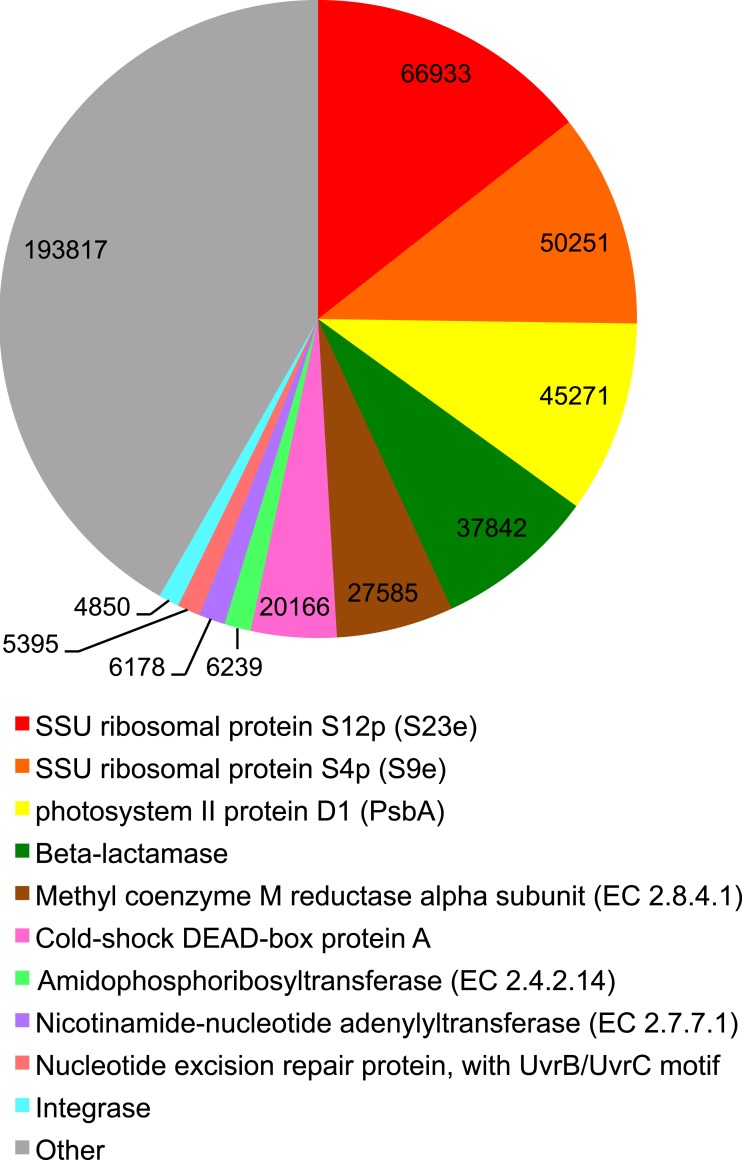
Top 10 expressed functions in the Landsort Deep sediment community. The 10 most abundant functions (SEED) in the sediment metatranscriptome that together added up to >50% of the total number of annotated transcripts. Numbers represent the absolute number of hits to the annotated function in the metatranscriptome.

**Figure 3:**
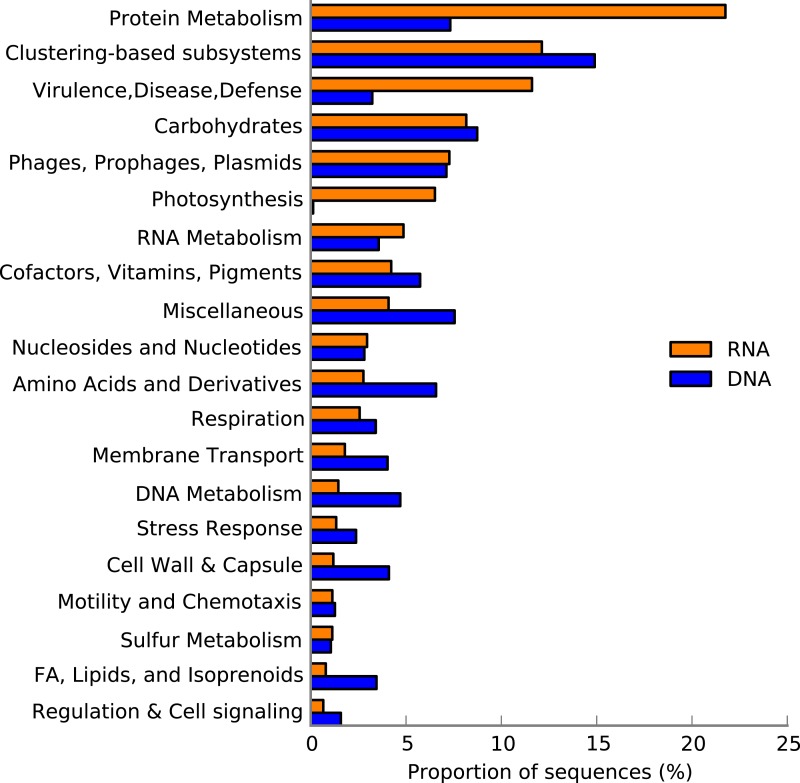
Expression of functions, SEED categories, in the Landsort Deep sediment community. Functional diversity at the transcriptional level with relative abundances of SEED categories (level 1) in the sediment metatranscriptome and metagenome, respectively. The 20 most abundant SEED categories of a total of 28 SEED categories in the metatranscriptome are shown and sorted from top to bottom based on high to low abundance.

We identified transcripts of genes both within metabolic (Fig. 1) and regulatory (Fig. S1) KEGG pathways in the sediment metatranscriptome. Regulatory pathways, especially genes associated with translation, i.e., *Ribosome* and *Aminoacyl-tRNA biosynthesis*, were extensively expressed (Fig. S1) and transcripts encoding ribosomal proteins were the most abundant (Fig. 2). Our results revealed major differences in transcript abundance among functions at the 1st level of SEED classification (Fig. 3). Out of the total 28 functions (SEED categories), the dominating 50% included *Protein-, RNA- and DNA metabolism* and *Nucleosides and Nucleotides* (Fig. 3). We found that a large portion of functions was expressed for both *Protein metabolism* (58%; 253 out of 438 genes expressed) and *Cell Division and Cell Cycle* (53%; 34 of 64 genes expressed) (Table S3).

### Chemotaxis, motility and adhesion

Results showed extensive expression of genes associated with *Motility and Chemotaxis* (*n* = 8, 316; 46 of 89 genes expressed) (Fig. 3, Fig. S1 and Table S3). Especially transcripts encoding flagellar proteins as flagellin protein FlaA (*n* = 1, 825) and flagellar biosynthesis protein FlhB (*n* = 1, 344) were highly abundant in the metatranscriptome whereas transcripts encoding chemotaxis proteins, such as the chemotaxis regulator proteins CheY (*n* = 57) and CheW (*n* = 53) were less abundant (Table S4). Furthermore, transcripts associated with *Adhesion* (*n* = 1, 437), particularly the cell-wall-anchored protein SasA (Table S4), were also identified in the metatranscriptome.

### Enzymatic hydrolysis of organic matter

This study reveals expression of genes encoding key enzymes for degradation of carbohydrates and polymers (i.e., within the SEED categories *Aminosugars, Polysaccharides* and *Glycoside hydrolase*; Table S5). Transcripts of enzymes in the latter two were overrepresented in Landsort Deep sediment (8,336 and 1,679 counts per million (CPM) for *Polysaccharides* and *Glycoside hydrolase* respectively) compared to other sediment metatranscriptomes (between 402 and 1412 CPM and 0 and 539 CPM respectively) (Fig. S2). We also identified transcripts for beta-hexosaminidase (EC 3.2.1.52) (*n* = 49) and chitinase (EC 3.2.1.14) (*n* = 35) necessary for chitin utilization (Table S5) and transcripts important for degradation of xyloglucan (i.e., hemicellulose) e.g., alpha-L- fucosidase (EC 3.2.1.51) (*n* = 37) (Table S5). For genes associated with the *Alpha-Amylase locus* two of three genes were expressed as transcripts, and for *Glycogen metabolism* six of nine genes were expressed (Table S5). Most of these transcripts were matched to glycogen phosphorylase (EC 2.4.1.1) (*n* = 152) (Table S5).

### Dissimilatory sulphate reduction and denitrification

Transcripts encoding all the necessary enzymes for dissimilatory sulphate reduction were identified, i.e., sulphate adenylyltransferase (Sat) EC 2.7.7.4, adenylylsulphate reductase (AprAB) EC 1.8.99.2 and sulphite reductase (DsrAB) EC 1.8.99.5 (Fig. S3 and Table S4). Moreover, genes encoding the complete sulphite-reduction associated complex DsrMKJOP were expressed (Table S4). We detected an incomplete denitrification pathway with transcripts encoding nitrate reductase (EC 1.7.99.4) and cytochrome cd1 nitrite reductase (EC 1.7.2.1) but with no transcripts encoding enzymes for nitric oxide reduction through nitrous oxide to nitrogen (Fig. S4).

### Fermentation

All fermentative pathways in the SEED classification were expressed to varying extent (36–67% of genes expressed), with the major portion of genes expressed for butanol biosynthesis and acetyl-CoA fermentation to butyrate (Table S5). The most abundant transcript (*n* = 1, 322) associated with fermentative processes encoded the enzyme pyruvate formate lyase (EC 2.3.1.54) (Fig. 1) followed by transcripts encoding acetolactate synthase EC 2.2.1.6 (*n* = 984) and butyryl-CoA dehydrogenase EC 1.3.99.2 (*n* = 115), respectively (Table S5).

### Methanogenesis and anaerobic methane oxidation

There was a high abundance of transcripts of the *mcrA* gene that encodes the alpha subunit of the methanogenic key enzyme, methyl coenzyme M reductase (Fig. 2) in Landsort Deep sediment, also in comparison to other sediment metatranscriptomes in which we did not detect any transcripts (Fig. S2).

Indeed, transcripts encoding subunits of all enzymes in complete methanogenic pathways from carbon dioxide, methanol and acetate were detected, even if not all subunits in all enzymes were detected (Fig. 1, Fig. S5 and Table S6). An incomplete pathway from trimethylamine to methane was also identified in the metatranscriptome (Table S6). Taxonomic analysis showed that transcripts specific for methane production from carbon dioxide were predominantly assigned to Methanosarcinales of the phylum Euryarchaeota, whereas transcripts for the other methanogenic pathways were assigned to different taxa including bacterial species (Table S6). Phylogenetic analysis of *mcrA* transcript sequences with those of selected methanogenic and ANME archaea revealed two types of *mcrA* genes, one that was most similar to *mcrA* genes of methanogenic Methanosarcina, and one that was most similar to *mcrA* genes of group e belonging to ANME-2a (Fig. S6). Transcripts were also identified (in the SEED annotation) for the particulate methane monooxygenase C subunit (*pmoC*) (Table S4) and taxonomically assigned to methanotrophic Methylococcaceae. The methanotrophic pathway from methane to carbon dioxide was, however, incomplete since transcripts only encoded formate dehydrogenase (EC 1.2.1.2) but no enzymes for oxidation of methanol through formaldehyde to formate.

### Resistance mechanisms and mobile genetic elements

Eight percent of the sediment metatranscriptome were beta-lactamase transcripts (Fig. 2). However, the beta-lactamase transcripts were traced to one single ORF flanked by regions 400 bp upstream and 100 bp downstream with no corresponding match to the diverse set of beta-lactamase genes found in the metagenome. Sequence analysis (BLAST) showed best match to a TEM-type beta-lactamase and beta-lactamases in different commercial expression and cloning vectors. From sequence alignments to the vectors used in our lab, we found no signs that the beta-lactamase transcripts were a result of contamination by any of our tools. Transcripts of cobalt-zinc-cadmium (*n* = 35) and arsenic (*n* = 9) resistance genes were detected (Table S4). Moreover, phage integrase transcripts were abundant (Fig. 2). A large portion of expressed genes associated with *Phage integration and excision* (46%; 6 of 13 genes expressed) and *Phage packing machinery* (58%; 7 of 12 genes expressed) (Table S5) was expressed. Transcripts of viral structural genes, such as major capsid proteins, were also present (Table S4). High abundances of integron integrase transcripts were identified (*n* = 1, 215) (Table S4) and integron integrase transcripts were overrepresented in the Landsort Deep sediment metatranscriptome (463 CPM) when compared to metatranscriptomes of Peru Margin sub-seafloor (9.87, 19.6, 0 and 0 CPM in the 5 m, 30 m, 70 m and 159 m samples respectively) (Orsi et al., 2013) and Arctic Jan Mayen vent field (0 CPM) (Urich et al., 2014) sediments (Fig. S2).

### Transcripts associated with photosynthesis

Surprisingly, our results showed a high abundance of transcripts annotated to the SEED category *Photosynthesis* (Fig. 3). The majority (>95%) of these transcripts, however, were annotated to a single gene, *psbA* encoding the D1 protein of the PSII P680 reaction centre (Fig. 1). In fact, *psbA* was the third most abundant transcript in the metatranscriptome (Fig. 2). Transcripts of *psbA* were unique for the Landsort Deep sediment metatranscriptome relative to the other sediment metatranscriptomes (Fig. S2) and could only be assigned at a very broad taxonomic level (“cellular organism” and “root” in the NCBI taxonomy) by the LCA algorithm (Huson et al., 2007). However, the best BLASTX hits to Landsort Deep *psbA* transcripts were sequences from eukaryotic algae (Fig. S7), predominantly diatoms (i.e., Bacillariophyta) that were abundant in the sediment rRNA (2% of total SSU rRNA, 10% of total LSU rRNA).

### Distribution of transcripts across taxa

**Figure 4:**
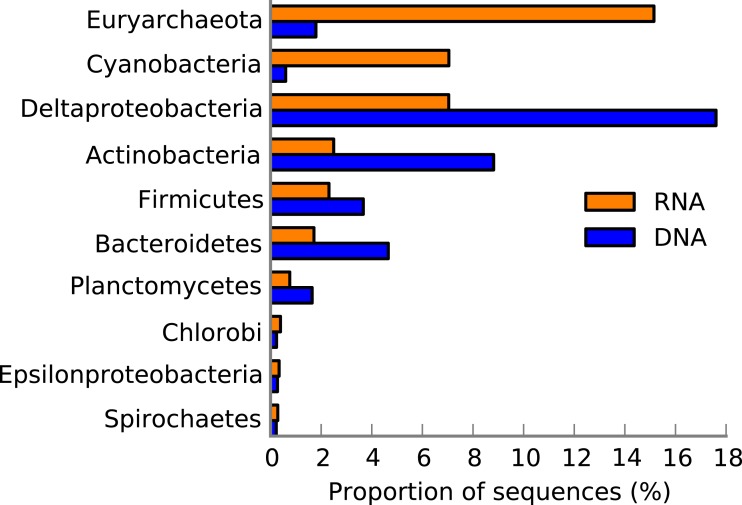
The 10 most abundant taxa in the Landsort Deep sediment community. Taxonomical distribution of functions (SEED categories) in the metatranscriptome and metagenome of Landsort Deep sediment. Only the 10 most abundant phyla or proteobacterial classes in the metatranscriptome are shown and sorted from top to bottom based on high to low abundance in the metatranscriptome.

**Figure 5:**
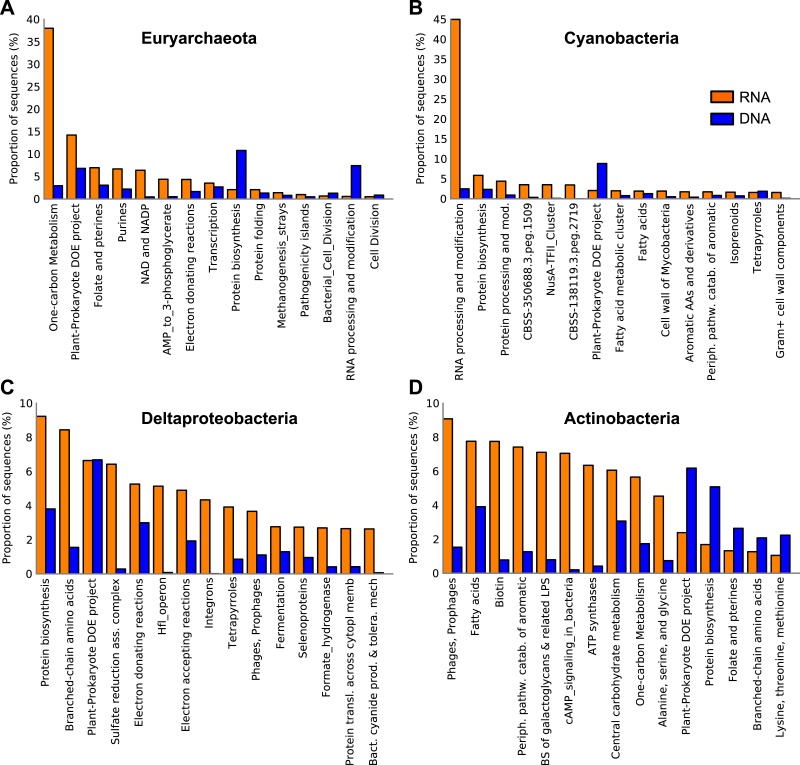
Expressed functions by the most abundant taxa in the Landsort Deep sediment metatranscriptome. Distribution of transcripts of the four most dominant phyla and proteobacterial classes in the Landsort Deep sediment metatranscriptome; (A) Euryarchaeota (B) Cyanobacteria (C) Deltaproteobacteria (D) Actinobacteria. The 15 most abundant SEED categories (level 2) in the (meta)transcriptome for each taxa are shown and sorted from left to right based on high to low abundance. Corresponding metagenomic (DNA) data is also presented. The *Plant-Prokaryote DOE project* is a miscellaneous SEED category comprising a diverse set of genes identified during investigation of plant-prokaryote interactions by a project at the Department of Energy (DOE), USA. CBSS-350688.3.peg.1509 and CBSS-138119.3.peg.2719 are clustering-based subsystems in which there is functional coupling and evidence that genes belong together, but with unknown functions. BS of galactoglycans & related LPS, Biosynthesis of galactoglycans and related lipopolysaccharides; Periph. pathw. catab. of aromatic, Peripheral pathways for catabolism of aromatic compounds.

Taxonomic analysis of protein coding genes and transcripts showed disconnect between the metagenome and metatranscriptome (Fig. 4). Euryarchaeota was the major taxon in the metatranscriptome (Fig. 4) in which it was 8-fold more abundant compared with the metagenome. A large portion (40%) of the euryarchaeal transcripts was associated with methanogenesis (see *One-carbon Metabolism*, Fig. 5A). At a more detailed taxonomic level, euryarchaeal methanogenic transcripts could be assigned to the family Methanosarcinaceae (40–50% of the total methanogenesis sequence reads). The most abundant transcript (38%) in the euryarchaeal transcriptome encoded methyl coenzyme M reductase alpha subunit (EC 2.8.4.1), followed by transcripts encoding amidophosphoribosyltransferase EC 2.4.2.14 (9%, see *Plant-Prokaryote DOE project* (miscellaneous SEED category), *Folate and Pterins* and *Purines*, Fig. 5A) and nicotinamide-nucleotide adenylyltransferase, NadM family EC 2.7.7.1 (9%, see *Plant-Prokaryote DOE project* and *NAD and NADP*, Fig. 5A) (Table S7).

Interestingly, Cyanobacteria was the second most abundant taxon in the Landsort Deep metatranscriptome, approximately 10-fold more abundant than in the metagenome (Fig. 4). More than 90% of the reads could only be assigned at phylum level but when assigned at lower ranks Nostocales (4%) and Chroococcales (2%) were most common. A major portion of the cyanobacterial transcripts was associated with *RNA processing and modification* (Fig. 5B). This was largely a reflection of the many transcripts (61% of cyanobacterial transcripts) encoding the cyanobacterial cold-shock DEAD-box protein A (Fig. 2, Table S7). Several transcripts were associated with *Protein biosynthesis* and *Protein processing and modification* including the second most abundant transcript that encoded the ribosomal protein S12p Asp88 methylthiotransferase (7%). Transcripts encoding the translation initiation factor 2 (5%) were the third most abundant in the cyanobacterial transcript pool (Table S7) (see *Protein biosynthesis*, *CBSS-350688.3.peg.1509, NusA-TFII cluster* and *CBSS-138119.3.peg.2719*, Fig. 5B).

Deltaproteobacteria was the third most abundant taxon among the transcripts (Fig. 4) and it was the most abundant taxon in the metagenome (Fig. 4). Desulfobacteraceae (40%) was the most common family within Deltaproteobacteria. Unlike Euryarchaeota and Cyanobacteria, the deltaproteobacterial transcriptome was distributed across several different functional categories, including *Sulfate reduction associated complex* (6%) and *Fermentation* (3%) (Fig. 5C). In addition, eighty percent of the total transcripts for dissimilatory sulphate reduction were assigned to Deltaproteobacteria. The most prevalent deltaproteobacterial transcripts encoded hydroxymethylglutaryl-CoA lyase EC 4.1.3.4 (12%, see *Branched-chain amino acids*, Fig. 5C), HflK protein (7%, see *Plant-Prokaryote DOE project* and *Hlf operon*, Fig. 5C) and glutamyl-tRNA synthetase (6%, see *Protein biosynthesis* and *Tetrapyrroles*, Fig. 5C) (Table S7).

Actinobacteria was also abundant (the 4th taxon) in the metatranscriptome, similarly to the metagenome, where it was second after Deltaproteobacteria (Fig. 4). Main functional categories of actinobacterial transcripts included *Fatty Acids, One-carbon metabolism, Central carbohydrate metabolism* and *Peripheral pathways for catabolism of aromatic compounds* (Fig. 5D). The three most abundant actinobacterial transcripts encoded integrase (16%, see *Phages, Prophages,*Fig. 5D), long chain fatty acid CoA ligase EC 6.2.1.3 (13%, see *Fatty Acids*, *Biotin*, *Peripheral pathways for catabolism of aromatic compounds*, Fig. 5D) and cAMP-binding protein (13%, see *cAMP signalling in bacteria* and *Biosynthesis of galactoglycans and related lipopolysaccharides*, Fig. 5D) (Table S7).

## Discussion

The Baltic Sea holds some of the largest oxygen depleted and sulphidic areas in the world (Diaz & Rosenberg, 2008). It is predominantly an effect of eutrophication with seasonal phytoplankton blooms and oxygen depleting heterotrophic respiration. Pelagic and coastal sediment microbial communities of the Baltic Sea have been comprehensively studied (e.g., Edlund et al., 2008; Dupont et al., 2014). However, the community of the habitat persistently exposed to oxygen depletion, the deepest point of the Baltic Sea, has previously received little attention (Thureborn et al., 2013). While biogeochemical analysis may reveal whether a microbiome responds to environmental conditions and contributes to key ecosystem functions it does not provide genetic information suggesting how this is accomplished. This study shows what genes of metabolic and regulatory pathways are expressed by the Landsort Deep sediment community. Results reveal that the community is active *in situ* expressing a large portion of the genome content detected in the metagenome (Fig. 1). The presence of transcripts, i.e., relatively unstable environmental mRNA, demonstrates that the deep trench sediment metagenome comprises living cells and not solely preserved DNA of dead cells deposited from the water column (Corinaldesi et al., 2011). These results further strengthen our previous metagenomic results (Thureborn et al., 2013). Moreover, the comparison with other metatranscriptomes from sediments, although limited by the presence of few such studies and inherent differences in sampling and laboratory procedures (Jones et al., 2015), highlights the special characteristics of the Landsort Deep sediment and the importance of future comparative work.

The eutrophication process, climate and the Baltic Sea bathymetry all influence the environmental conditions at Landsort Deep. Euxinic conditions with high sediment deposition rates, anoxic sediment and H_2_S in the overlying waters (Lepland & Stevens, 1998; Leipe et al., 2011) were confirmed at the time of sampling by a high concentration of organic carbon in the sediment (79.0 mg/L DOC) and sulphidic bottom water (16.3 µmol/L H_2_S) (Table S2). Landsort Deep metatranscriptomic results reflect these environmental conditions and propose a sediment community capitalising on the deposited organic carbon, possibly with a life strategy of sensing and attaching to the carbon source (Keyhani & Roseman, 1999). For example, transcripts encoding proteins involved in motility and attachment indicate that some of the Landsort Deep microorganisms move toward or attach to surfaces and substrates, such as aggregates of cells and organic debris from the water column deposited at the sediment surface (Fig. 3, Fig. S1 and Table S3). Once attached, the microorganisms could convert the organic matter to less complex compounds. Indeed, expressions of several genes encoding key enzymes for degradation of carbohydrates and polymers (Table S5) suggest that the sediment community mineralises the organic matter. For example, transcripts for chitinase support that specifically chitin is hydrolysed into oligo- or dimers and that this polymer, present in debris derived from zooplankton and invertebrates (Gooday, 1990), is a metabolic resource for the Landsort Deep sediment community. The expressions of glycoside hydrolase genes and several other genes for enzymatic hydrolysis of carbon polymers imply that there are substrates available for further mineralisation in the sediment through anaerobic heterotrophy.

Dissimilatory sulphate reduction was an anaerobic heterotrophic pathway of which the complete gene suite was expressed (Fig. S3). Metabolism with sulphate as electron acceptor in heterotrophy was also supported by the highly sulphidic environment of Landsort Deep (Table S2). Organic matter, which has been mineralised into smaller organic carbon compounds—e.g., volatile fatty acids (VFA) such as acetate, lactate, formate and propionate—may serve as electron donors in sulphate reduction. These smaller organic compounds, at a concentration of 79.0 mg/L at Landsort Deep (Table S2), are primarily products from microbial fermentation of monomers and oligomers (Holmer & Storkholm, 2001; Finke & Jorgensen, 2008). Landsort Deep sulphate reducers hence depend on other community members, i.e., fermenters, to provide electron donors, in this study corroborated by a high proportion of expressed genes within fermentative pathways (Table S5). Since the sediment community expressed all fermentative pathways, sulphate reducers probably have access to the necessary carbon substrates. Although the presence of transcripts does not confirm enzymatic activity, these results are consistent with active heterotrophic mineralisation of dissolved organic matter through fermentation and dissimilatory sulphate reduction in the Landsort Deep sediment.

Methanogenesis was one of the major ecosystem processes identified in the metatranscriptome (Fig. 1) which corresponds well with the high methane concentrations (Schmale et al., 2010; Jakobs et al., 2013) in the anoxic water of Landsort Deep and our previous metagenome results (Thureborn et al., 2013). All four methanogenic pathways, from carbon dioxide, acetate, methanol and methylamines, respectively, were present according to transcript information (Table S6). Moreover, abundance data and taxonomic information of transcripts encoding methanogenic enzymes suggest that methane production from carbon dioxide by Methanosarcinales is the predominant methanogenic pathway in the Landsort Deep sediment (Table S6). Interestingly, the *mcrA* gene, expressed in the Landsort Deep sediment, was not detected in the Peru Margin and Arctic Jan Mayen sediments (Fig. S2). The absence of *mcrA* transcripts in the Peru Margin sediment metatranscriptomes has been suggested to be a consequence of low archaeal expression and a masking of the *mcrA* expression by housekeeping genes (Orsi et al., 2013). The high expressions of euryarchaeal genes in general, and *mcrA* in particular, in Landsort Deep sediment hence suggest comparatively high methanogenic activity at this site. Furthermore, our results indicate that active anaerobic methane oxidation occurs in the anoxic sediment of Landsort Deep. It could be possible both by reverse methanogenesis by Archaea in the ANME group (Fig. S6) in cooperation with coexisting sulphate reducing Deltaproteobacteria (i.e., Desulfobacteraceae) (Fig. 4) (Hallam et al., 2004; Knittel & Boetius, 2009) and by bacterial anaerobic methane oxidation by Methylococcaceae using available nitrate as electron acceptor (Table S2) (Ettwig et al., 2010). *In situ* oxidation of produced methane hence potentially restricts methane release to the hydrosphere. These results are supported by a recent study, which demonstrated bacterial aerobic methane oxidation in the redox zone of the Landsort Deep water column but also proposed that methane was oxidised anaerobically in the anoxic deep water (Jakobs et al., 2013).

**Figure 6:**
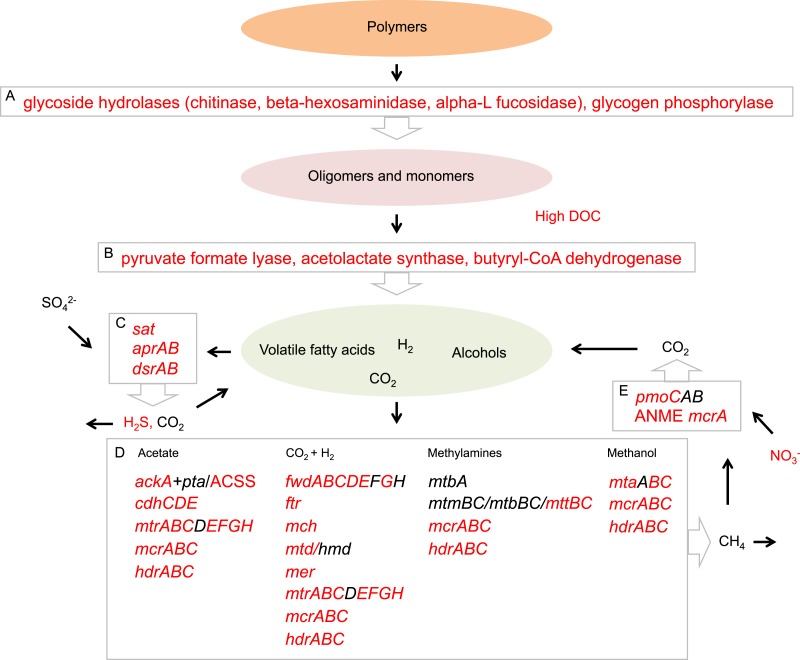
Proposed Landsort Deep sediment community interactome. A model inferred from transcripts showing putative interactions between metabolic processes involved in organic matter mineralisation and carbon transformation in the Landsort Deep sediment community. (1) Enzymatic hydrolysis represented by the most abundant enzyme transcripts. (2) Fermentation represented by the most abundant enzyme transcripts. (3) Dissimilatory sulphate reduction—transcripts were identified for all genes necessary for this process (4) Methanogenesis—transcripts of all genes necessary for methanogenic pathways using the different substrates acetate, CO_2_ + H_2_, methylamines and methanol, respectively. (5) Anaerobic methane oxidation represented by bacterial particulate methane monooxygenase (*pmoCAB*) and ANME methyl coenzyme M reductase A (*mcrA)*. Red colour indicates detected environmental parameters and identified gene transcripts in Landsort Deep sediment.

Energy metabolism in the sediment community of Landsort Deep, as inferred from expressed transcripts, appears to a large extent comprise the processes fermentation, methanogenesis, sulphate reduction and methane oxidation (Fig. 6). Drawing on these results, the Landsort Deep sediment methanogens and sulphate reducers may use products of fermentation as electron donors acting as sinks for hydrogen and organic acids. This may increase the fermentation rate, since concentrations of metabolites stay low, and improve the supply of substrates for respiring bacteria (Fenchel & Finlay, 1995). Organic acids would otherwise build up in the immediate environment and potentially reduce the efficiency of fermentation (Fenchel & Finlay, 1995). Methanogenic archaea, fermenting bacteria and anaerobic respiring bacteria may well make up synergistic assemblages, an “interactome”, to which anaerobic methane oxidisers affiliate to capitalise on the methane (Fig. 6). Importantly, these results show that the microbial community expresses an array of metabolic enzyme genes that are crucial for transformation of the organic matter deposited in the sediment of Landsort Deep.

A characteristic feature of the Landsort Deep and other anoxic sediment metagenomes was the high abundance of integrons (Thureborn et al., 2013). The high abundance of transcripts encoding integron integrases detected in this study demonstrates that the integron genes of the Landsort Deep sediment are indeed expressed (Table S4). Further- more, transcripts encoding integron integrases are overrepresented in the metatranscriptome of Landsort Deep sediment in comparison with other sediments (Fig. S2). Integrons carry a large pool of adaptive genes with a potential to facilitate acquisition and recombination of foreign DNA into microbial genomes that may improve the recipient’s environmental fitness (Labbate, Case & Stokes, 2009), and integron recombination events have been found to be induced by environmental stress (Guerin et al., 2009). Notably, prevalence of integrons has been shown to increase with the degree of environmental pollution (Hardwick et al., 2008; Wright et al., 2008; Rosewarne et al., 2010) and the class 1 integron-integrase gene has been suggested as a proxy for anthropogenic pollution (Gillings et al., 2015). The comparatively high abundance of integron integrase transcripts in Landsort Deep sediment possibly reflects the high concentrations of pollutants at this site (HELCOM, 2010). Interestingly, Landsort Deep sediment (the same sample as in this study) has been shown to be a hot spot of IncP alpha plasmids, mobile genetic elements that may contribute to bacterial survival in polluted environments (Dealtry et al., 2014).

Environmental conditions at Landsort Deep, i.e., permanent darkness and anoxia at approximately 460 m water depth, are not expected to favour activity connected with photosynthesis. Surprisingly, our results show a high abundance of eukaryotic transcripts encoding the D1 protein of the PSII P680 reaction centre (*psbA*), particularly from diatoms (Fig. 2, Fig. S7). Expression of *psbA* in sunlit anoxic sediments was recently observed for oxygenic photosynthesis (Spain et al., 2015). However, high abundance of *psbA* transcripts in a permanently dark environment as the Landsort Deep sediment may be a consequence of how the PsbA protein synthesis is regulated in eukaryotic micro-algae. According to a previous study, stable pool of *psbA* transcripts accumulates during darkness as a result of constitutive transcription and strong regulation of protein biosynthesis at the level of translation initiation (Mulo, Sakurai & Aro, 2012). Moreover, constitutive expression of this gene has been shown in diatoms (Nymark et al., 2013). A possible scenario is that *psbA* transcripts at Landsort Deep remain in micro algae reaching the anoxic sediment after the spring phytoplankton bloom (Ploug & Grossart, 2000; Sarthou et al., 2005) that peaked in April just prior to sampling and in which diatoms are the major constituents (Kaitala, Hällfors & Maunula, 2010).

Another unexpected finding was the high abundance of transcripts assigned to Cyanobacteria (Fig. 4). These results, however, are corroborated by the high abundance of Cyanobacteria in the rRNA (P Thureborn, 2015, unpublished data) and support our previous finding of Cyanobacteria in the Landsort Deep sediment metagenome (1.6% of protein coding genes) (Thureborn et al., 2013). Cyanobacteria are capable of fermentative metabolism under dark and anoxic conditions (Stal & Moezelaar, 1997). However, the lack of evidence for cyanobacterial fermentative metabolism in the metatranscriptome (Table S7) suggests other reasons that better may explain the presence of Cyanobacteria in the Landsort Deep sediment. Cells may originate from the upper water column (Funkey et al., 2014), experience environmental stress and maintain non-growth functions (Schimel, Balser & Wallenstein, 2007) in the cold, dark and anoxic sediment. This is consistent with high expression of the cold-shock DEAD-box protein A (Fig. 2, Table S7), which has been shown to be involved in ribosome biogenesis, RNA turnover and translation initiation and cyanobacterial adaptation to low temperature (Chamot et al., 1999; Redder et al., 2015). These are all important processes in adaptation to a changing environment and stress response. Studies have also shown that Synechocystis DEAD-box RNA helicase transcripts accumulate when the electron transport chain is reduced (Patterson-Fortin, Colvin & Owttrim, 2006), opening for the suggestion that abundance of these transcripts at deep sea sediments reflects RNA redox regulation. Our finding of cyanobacterial transcripts in the Landsort Deep anoxic sediment merits further studies on cyanobacterial physiology during dark anoxic conditions and the broader role of Cyanobacteria in the Baltic Sea carbon transformation.

In conclusion, analyses of environmental mRNA and DNA revealed that the diverse sediment microbiome of Landsort Deep expressed large portions of its functional capacity. The metatranscriptome and environmental data indicate a sediment community active in capitalising on organic matter through anoxic mineralisation and carbon transformation, possibly through synergistic interactions between fermenters, sulphate reducers, methanogens and methane oxidisers (Fig. 6). With the prospect of a progression of anoxic conditions in the Baltic Sea, our results contribute information for future efforts to foresee microbial ecosystem functions in a scenario in which euxinic conditions prevail. Furthermore our results advance the understanding, at transcriptional level, of microbial ecosystem functions in deep water anoxic sediments.

## Supplemental Information

10.7717/peerj.1593/supp-1 Figure S1 Expression of genes in regulatory pathways (KEGG) in the Landsort Deep sediment community Landsort Deep sediment metatranscriptome mapped to KEGG regulatory pathways in iPATH v2 (Yamada et al., 2011). Red indicates that the element of the pathway was present in the metatranscriptome.

10.7717/peerj.1593/supp-2 Figure S2 Comparison of expressed functions in Landsort Deep sediment metatranscriptome with other sediment metatranscriptomes Relative abundance of transcripts (percent of total metatranscriptome) for integron integrases, photosystem II protein D1, methyl coenzyme M reductase alpha subunit, glycoside hydrolases, polysaccharides, respectively, in sediment metatranscriptomes of Landsort Deep, Peru Margin- (Orsi et al., 2013) and Arctic Jan Mayen Vent field (Urich et al., 2014).

10.7717/peerj.1593/supp-3 Figure S3 Transcripts within sulfur metabolism, KEGG map Enzymes or genes with ≥1 hit in the Landsort Deep sediment metatranscriptome are coloured in red.

10.7717/peerj.1593/supp-4 Figure S4 Transcripts within nitrogen metabolism, KEGG map Enzymes or genes with ≥1 hit in the Landsort Deep sediment metatranscriptome are coloured in red.

10.7717/peerj.1593/supp-5 Figure S5 Transcripts within methane metabolism, KEGG map Enzymes or genes with ≥1 hit in the Landsort Deep sediment metatranscriptome are coloured in red.

10.7717/peerj.1593/supp-6 Figure S6 Network analysis of *mcrA* genes Network analysis of sequences of *mcrA* gene transcripts from Landsort Deep sediment and selected *mcrA* genes from methanogenic and ANME archaea, with respective GenBank accession number. Analysis was performed using ClustalW alignment and the Neighbor-Net (Bryant & Moulton, 2004) algorithm with uncorrected p distances implemented in SplitsTree4 (Huson & Bryant, 2006). Red labels indicate methanogenic *mcrA* transcripts; blue labels indicate ANME-2a *mcrA* transcripts. The bar represents uncorrected p distances.

10.7717/peerj.1593/supp-7 Figure S7 Taxonomic analysis of *psbA* transcripts in Landsort Deep sediment Taxonomic affiliation of the 25 most abundant photosystem II protein D1, *psbA* transcripts identified in the Landsort Deep sediment metatranscriptome comprising >90% of all reads assigned to *psbA*. Transcripts of the *psbA* gene were aligned against the NCBI NR database using BLASTX and subsequently analysed in MEGAN (Huson et al., 2011) using best hit as criteria. Ass, numbers of transcripts assigned to node; Sum, number of transcripts assigned at lower nodes. Four transcripts could only be assigned at the level of Eukaryota because they showed identical bit score to more than one eukaryotic organism.

10.7717/peerj.1593/supp-8 Table S1 Sequencing and annotation statistics Numbers from Illumina HiSeq 2000 sequencing and annotation of the Landsort Deep sediment microbiome cDNA and DNA.

10.7717/peerj.1593/supp-9 Table S2 Environmental parameters at Landsort Deep Environmental parameters determined at Station BY31, Landsort Deep, in the Baltic Sea on the 21st of April 2010, concurrently with sampling of the sediment for metagenomic and metatranscriptomic analyses.

10.7717/peerj.1593/supp-10 Table S3 Number of functions expressed within SEED level 1 categories The number of functions within SEED categories (level 1) that were present (≥1 hit) in the Landsort Deep sediment metatranscriptome and metagenome, respectively. The SEED categories are sorted from top to bottom based on high to low portion of functions expressed (i.e., number of functions in metatranscriptome/number of functions in the metagenome).

10.7717/peerj.1593/supp-11 Table S4 Abundance of all transcribed genes in the Landsort Deep sediment community SEED functions identified in the metatranscriptome and metagenome, respectively.

10.7717/peerj.1593/supp-12 Table S5 Functions expressed within selected SEED level 3 categories Functions within selected SEED categories (level 3) that were detected in the Landsort Deep sediment metatranscriptome and metagenome, respectively.

10.7717/peerj.1593/supp-13 Table S6 Abundance of transcribed methanogenic genes in the Landsort Deep sediment community Summary of KEGG functions associated with methanogenesis that were detected in the metatranscriptome and metagenome, respectively. Functions specific for the three different methanogenic pathways: CO_2_-, acetate-, methanol and methylamine to CH_4_, are highlighted in red, green, blue and orange, respectively. Taxonomic distribution of functions is expressed as percentage of the total sequences annotated to the function in the metatranscriptome.

10.7717/peerj.1593/supp-14 Table S7 All expressed functions of the most abundant taxa in the Landsort Deep sediment community SEED functions identified for Euryarchaeota, Cyanobacteria, Deltaproteobacteria and Actinobacteria in the metatranscriptome and metagenome, respectively.

## Additional Information and Declarations

### DNA Deposition

The following information was supplied regarding the deposition of DNA sequences: Sequence data were deposited at the European Nucleotide Archive (ENA) under the project PRJEB6616 with sample accession numbers ERS485200 (total cDNA), ERS485201 (mRNA enriched cDNA) and ERS485202 (DNA).

### Data Availability

The following information was supplied regarding data availability: GitHub, m5nr2annot and organisms2lca, https://github.com/erikrikarddaniel/environmentmicrobedb-tools.
