# Supplementary material for: Reconstructing ecosystem functions of the active microbial community of the Baltic Sea oxygen depleted sediments
Source: PeerJ. 2016 Jan 19;4:e1593. doi: 10.7717/peerj.1593 (PMC4730985; doi:10.7717/peerj.1593)
Supplement: Figure S1 — Landsort Deep sediment metatranscriptome mapped to KEGG regulatory pathways in iPATH v2 (Yamada et al., 2011). Red indicates that the element of the pathway was present in the metatranscriptome. [file peerj-04-1593-s001.pdf]

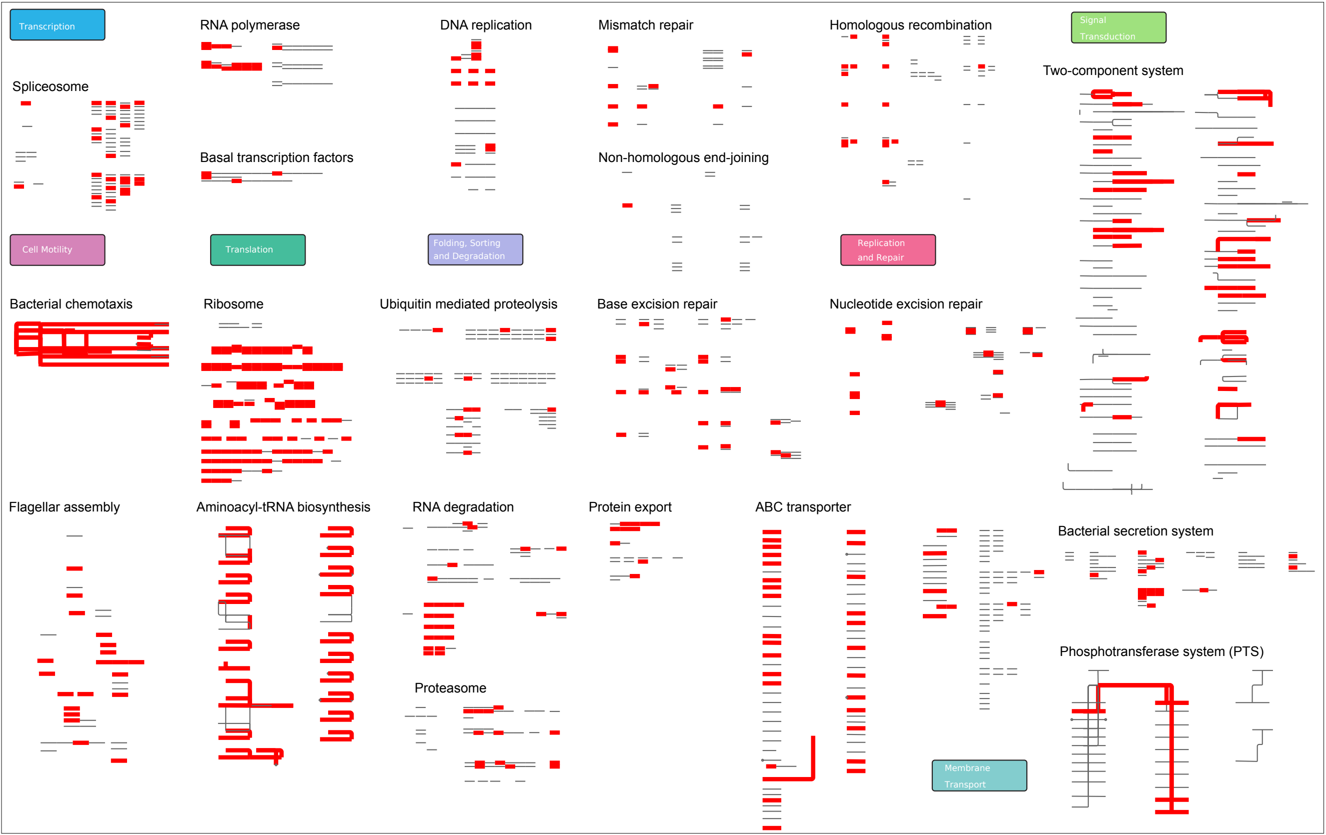

Transcription
RNA polymerase
DNA replication
Mismatch repair
Homologous recombination
Signal Transduction
Spliceosome
Two-component system
Basal transcription factors
Non-homologous end-joining
Cell Motility
Translation
Folding, Sorting and Degradation
Replication and Repair
Bacterial chemotaxis
Ribosome
Ubiquitin mediated proteolysis
Base excision repair
Nucleotide excision repair
Flagellar assembly
Aminoacyl-tRNA biosynthesis
RNA degradation
Protein export
ABC transporter
Bacterial secretion system
Phosphotransferase system (PTS)
Proteasome
Membrane Transport
