# Supplementary figures and images for: Reconstructing ecosystem functions of the active microbial community of the Baltic Sea oxygen depleted sediments
Source: PeerJ. 2016 Jan 19;4:e1593. doi: 10.7717/peerj.1593 (PMC4730985; doi:10.7717/peerj.1593)

# Comparison of expressed functions in Landsort Deep sediment with other sediments

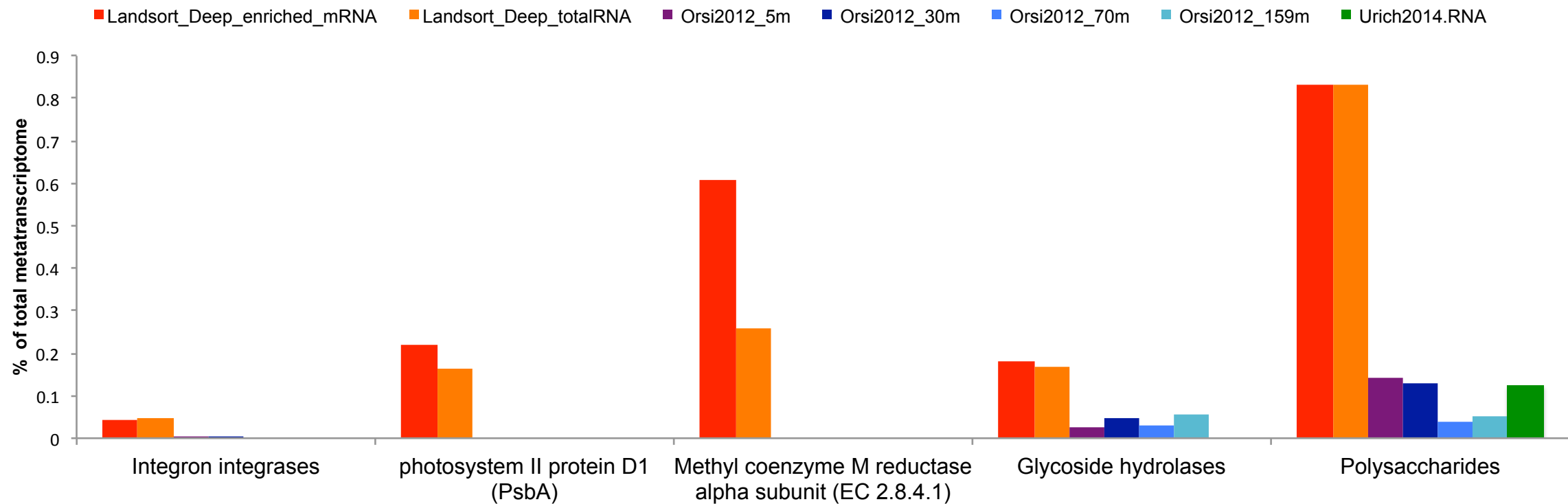

Supplement: Figure S2 — Relative abundance of transcripts (percent of total metatranscriptome) for integron integrases, photosystem II protein D1, methyl coenzyme M reductase alpha subunit, glycoside hydrolases, polysaccharides, respectively, in sediment metatranscriptomes of Landsort Deep, Peru Margin- (Orsi et al., 2013) and Arctic Jan Mayen Vent field (Urich et al., 2014). [file peerj-04-1593-s002.pdf]

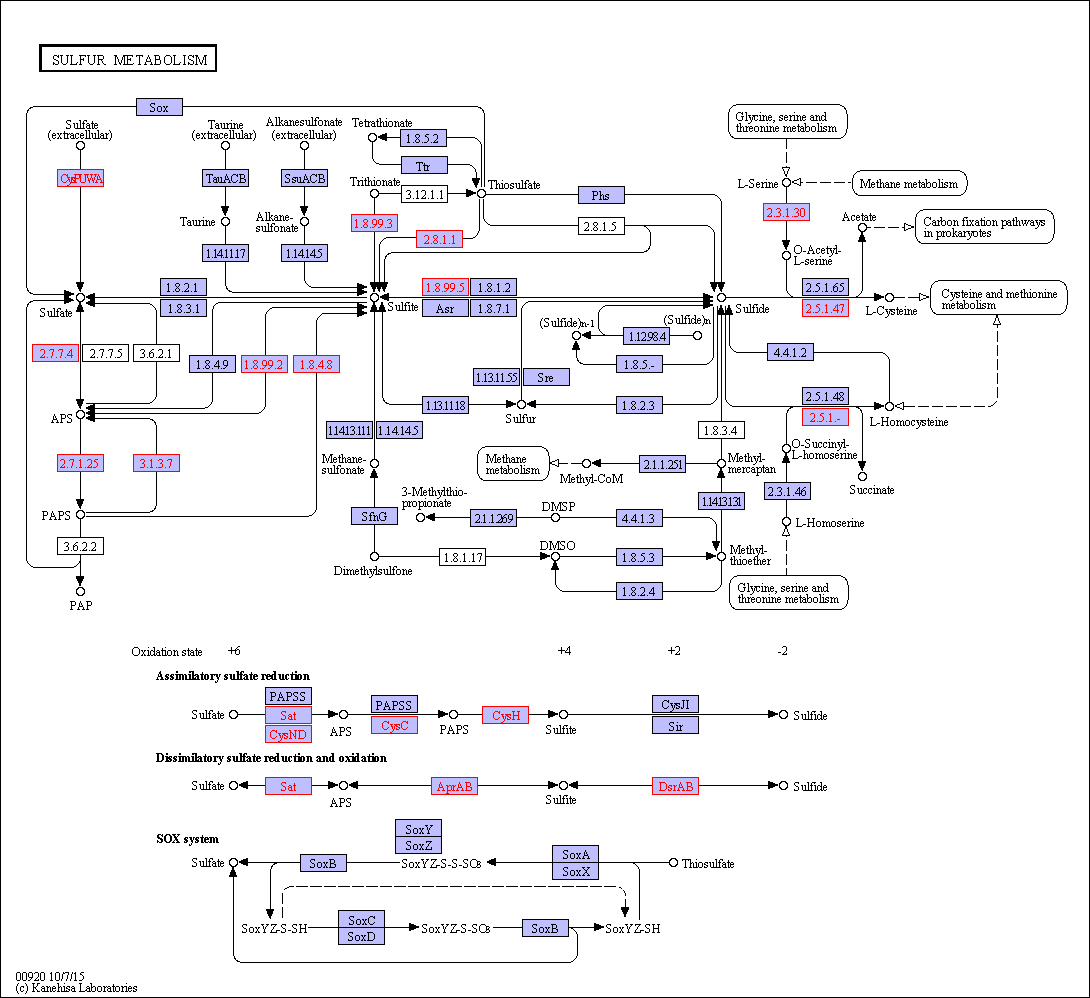

Supplement: Figure S3 — Enzymes or genes with ≥1 hit in the Landsort Deep sediment metatranscriptome are coloured in red. [file peerj-04-1593-s003.png]

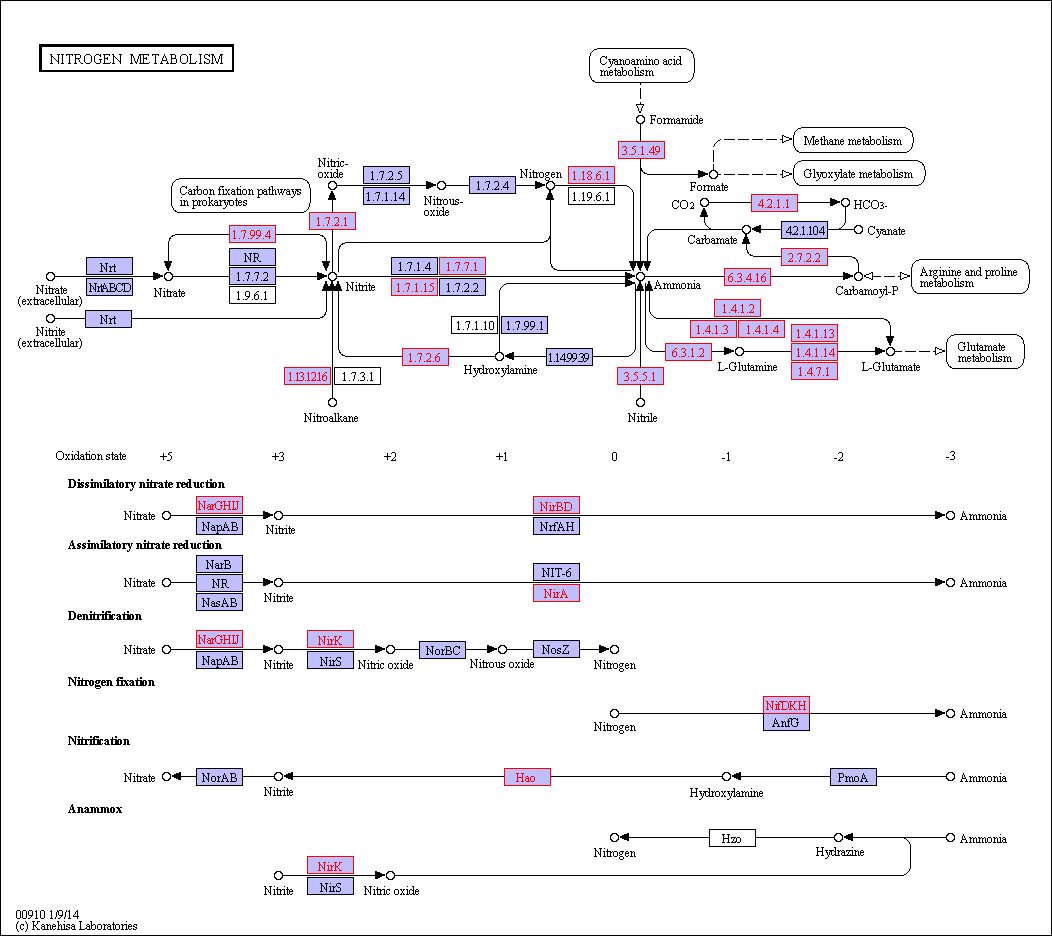

Supplement: Figure S4 — Enzymes or genes with ≥1 hit in the Landsort Deep sediment metatranscriptome are coloured in red. [file peerj-04-1593-s004.png]

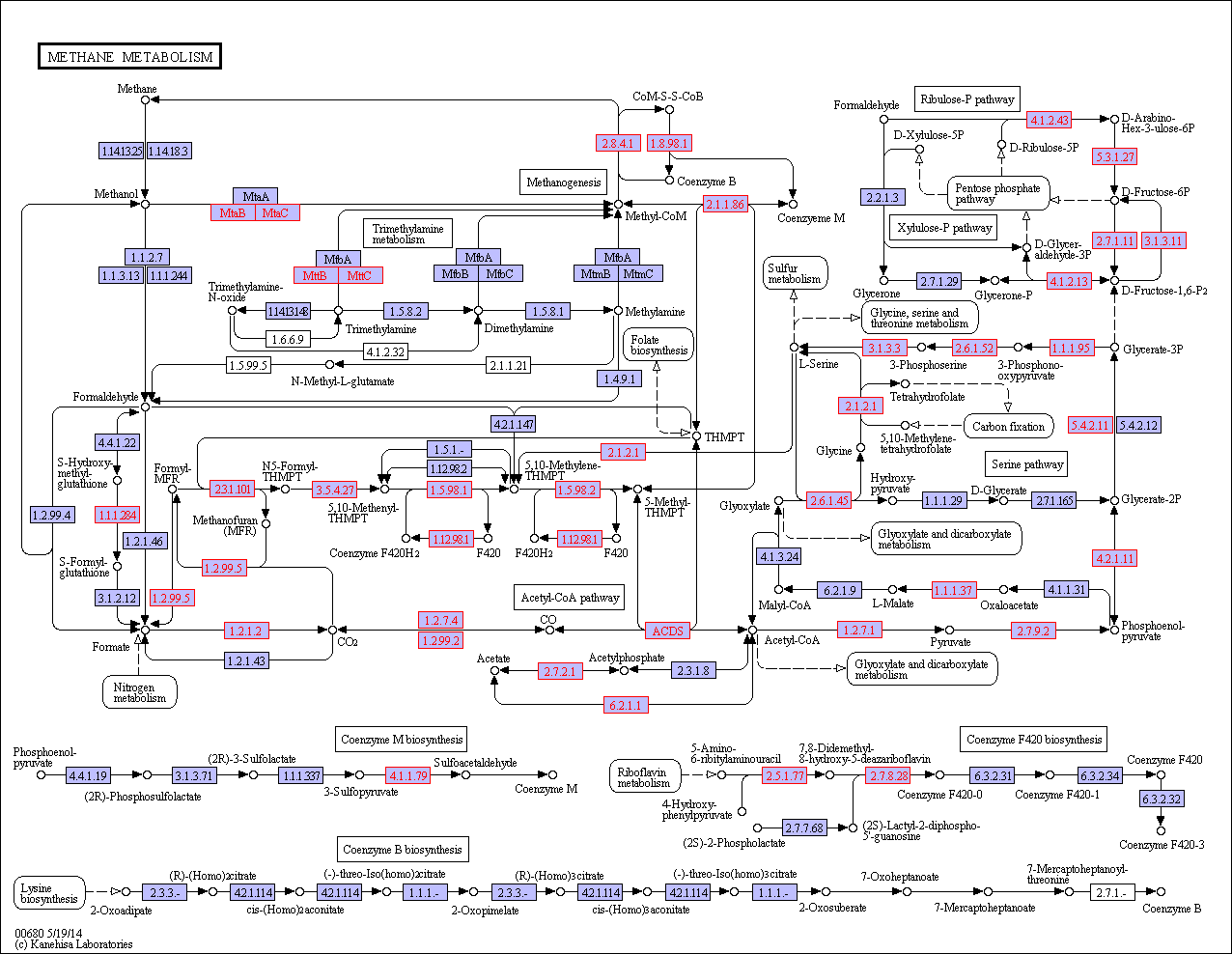

Supplement: Figure S5 — Enzymes or genes with ≥1 hit in the Landsort Deep sediment metatranscriptome are coloured in red. [file peerj-04-1593-s005.png]

0.1

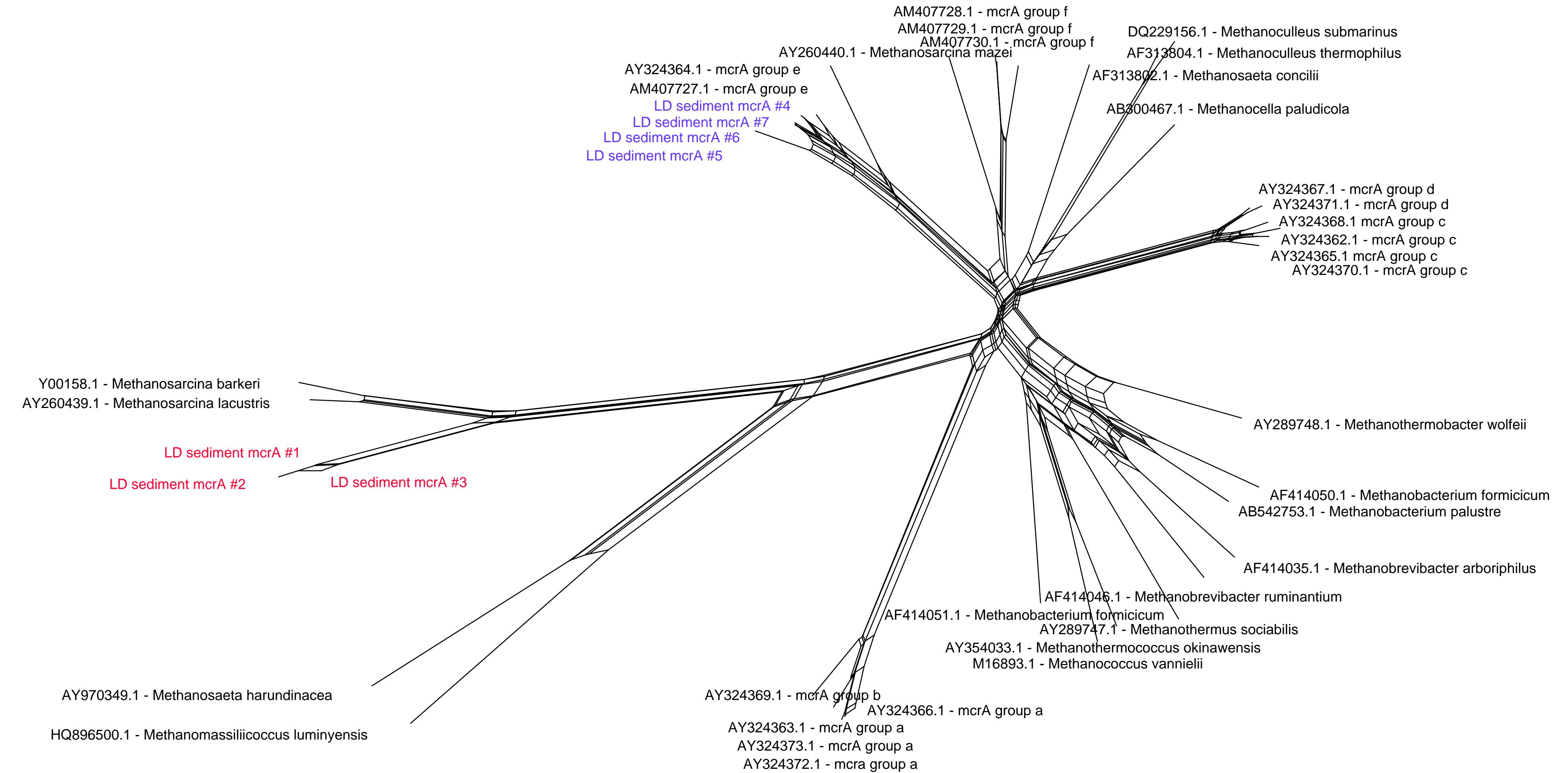

Supplement: Figure S6 — Network analysis of sequences of mcrA gene transcripts from Landsort Deep sediment and selected mcrA genes from methanogenic and ANME archaea, with respective GenBank accession number. Analysis was performed using ClustalW alignment and the Neighbor-Net (Bryant & Moulton, 2004) algorithm with uncorrected p distances implemented in SplitsTree4 (Huson & Bryant, 2006). Red labels indicate methanogenic mcrA transcripts; blue labels indicate ANME-2a mcrA transcripts. The bar represents uncorrected p distances. [file peerj-04-1593-s006.pdf]

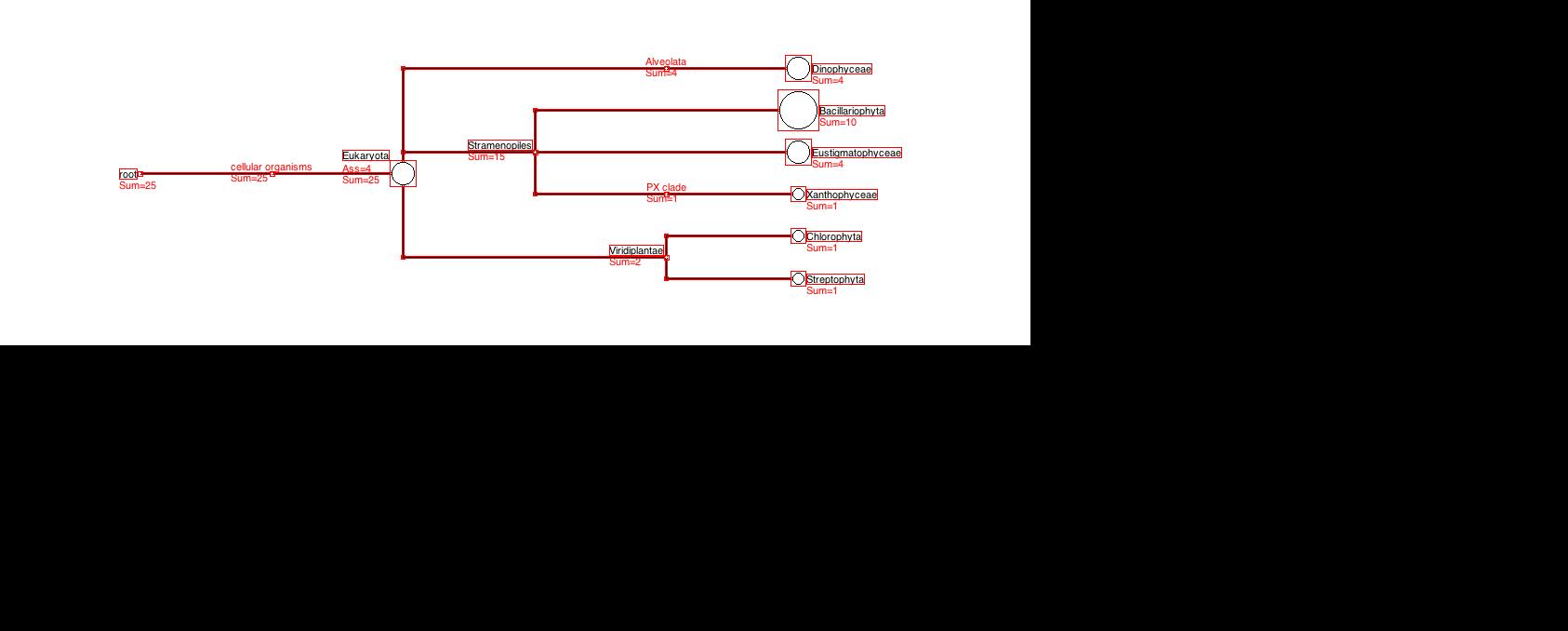

Supplement: Figure S7 — Taxonomic affiliation of the 25 most abundant photosystem II protein D1, psbA transcripts identified in the Landsort Deep sediment metatranscriptome comprising >90% of all reads assigned to psbA. Transcripts of the psbA gene were aligned against the NCBI NR database using BLASTX and subsequently analysed in MEGAN (Huson et al., 2011) using best hit as criteria. Ass, numbers of transcripts assigned to node; Sum, number of transcripts assigned at lower nodes. Four transcripts could only be assigned at the level of Eukaryota because they showed identical bit score to more than one eukaryotic organism. [file peerj-04-1593-s007.jpg]
