# Supplementary material for: Reconstructing ecosystem functions of the active microbial community of the Baltic Sea oxygen depleted sediments
Source: PeerJ. 2016 Jan 19;4:e1593. doi: 10.7717/peerj.1593 (PMC4730985; doi:10.7717/peerj.1593)
Supplement: Table S1 — Numbers from Illumina HiSeq 2000 sequencing and annotation of the Landsort Deep sediment microbiome cDNA and DNA. [file peerj-04-1593-s008.doc]

|  | **cDNA** | |  |
| --- | --- | --- | --- |
|  | **Total RNA** | **mRNA enriched** | **DNA** |
| Total number of Illumina HiSeq 2000 paired- end sequence reads (2 x 100 bp) | 95,232,313 | 92,143,054 | 106,080,777 |
| Number of paired-end sequence reads (2 x 100 bp) after quality filter and removal of rRNA sequences (only for cDNA) | 42,660,234 | 47,771,191 | 98,108,014 |
| Number of predicted genes | 429,162 | | 3,176,262 |
| Number of predicted genes with KEGG annotation | 15,109 | | 357,038 |
| Number of sequence reads mapping to gene with KEGG annotation | 590,366 | | 2,966,774 |
| Number of predicted genes with SEED annotation | 29,964 | | 618,715 |
| Number of sequence reads mapping to gene with SEED annotation | 5,099,614 | | 12,199,218 |
